# Induction of defense-related enzymes and enhanced disease resistance in maize against *Fusarium verticillioides* by seed treatment with *Jacaranda mimosifolia* formulations

**DOI:** 10.1038/s41598-020-79306-x

**Published:** 2021-01-08

**Authors:** Rabia Naz, Asghari Bano, Asia Nosheen, Humaira Yasmin, Rumana Keyani, Syed Tahir Abbas Shah, Zahid Anwar, Thomas H. Roberts

**Affiliations:** 1 Department of Biosciences, COMSATS University, Park Road, Chak Shahzad, Islamabad, Pakistan; 2 Department of Biosciences, University of Wah, Wah Cantt, Pakistan; 3 Department of Computer Science, COMSATS University Islamabad, Vehari Campus, Islamabad, Pakistan; 4 Plant Breeding Institute, Sydney Institute of Agriculture, University of Sydney, Sydney, NSW 2006 Australia

**Keywords:** Plant sciences, Plant immunity, Plant molecular biology, Plant physiology, Plant stress responses

## Abstract

*Fusarium verticillioides* is an important fungal pathogen of maize, causing stalk rot and severely affecting crop production. The aim of this study was to characterize the protective effects of formulations based on *Jacaranda mimosifolia* leaf extracts against *F. verticillioides* in maize*.* We compared different seed treatments comprising *J. mimosifolia* extracts, chemical fungicide (mefenoxam) and salicylic acid to modulate the defense system of maize host plants. Both aqueous and methanolic leaf extracts of *J. mimosifolia* (1.2% w/v) resulted in 96–97% inhibition of mycelial growth of *F. verticillioides*. While a full-dose (1.2%) extract of *J. mimosifolia* provided significant protective effects on maize plants compared to the inoculated control, a half-dose (0.6% w/v) application of *J. mimosifolia* in combination with half-strength mefenoxam was the most effective treatment in reducing stalk rot disease in pot and field experiments. The same seed treatment significantly upregulated the expression of genes in the leaves encoding chitinase, glucanase, lipid transfer protein, and pathogenesis-related proteins PR-1, PR-5 and PR-10, 72 h after inoculation. This treatment also induced the activities of peroxidase, polyphenol oxidase, protease, acid invertase, chitinase and phenylalanine ammonia lyase. We conclude that seed pre-treatment with *J. mimosifolia* extract with half-strength chemical mefenoxam is a promising approach for the management of stalk rot in maize.

## Introduction

Among the fungal diseases of maize (*Zea mays*), the most destructive is stalk rot, the causal agent of which is *Fusarium verticillioides* (synonym, *F. moniliforme*; teleomorph, *Gibberella moniliformis*), a cosmopolitan ascomycete that is consistently associated with maize worldwide^1^. *F. verticillioides* causes ear rot, kernel rot, stalk rot, seedling blight, seed rot, wilt and stunt^2,3^. Infected maize plants become distorted, resulting in losses of 10–35% in yield^4^. Stalk rot caused by *F. verticillioides* is the most prevalent disease of maize in various regions of Pakistan^5^.

Application of specific exogenous factors, such as the plant growth regulator salicylic acid (SA), can lead to rapid and coordinated activation of plant defense genes, with the corresponding gene products increasing resistance to pathogen attack^6–8^*.* This is a promising approach to reduce the dependence on chemical fungicides to control fungal diseases of crops^9^. SA is involved in signal transduction that induces the expression of both specific enzymes that catalyze reactions to form defense compounds such as polyphenols, as well as pathogenesis-related (PR) proteins, some of which have non-enzymatic functions^10,11^.

Currently, various bacterial species, as well as plant extracts, are being used as control agents against soil-borne phytopathogens such as *F. verticillioides*^12^. The bacteria *Burkholderia* spp., *Bacillus amyloliquefaciens* and *Enterobacter hormaechei* are reported to stimulate plant growth and suppress disease instigated by *F. verticillioides* in maize^3,13^. Plant extracts from *Azadirachta indica* and *Vernonia amygdaline* have been used for the control of *Fusarium moniliforme* (*F. verticilloides*)^14–16^. The use of plant-based biofungicides provides many distinctive benefits to growers, as they degrade rapidly, have very short pre-harvesting intervals, and reduce the risk of residual effects on food. Plant extracts used as fungicides are eco-friendly, and can be fast-acting, non-pollutive and potentially cost-effective compared to chemical fungicides^17^.

The in vitro antifungal potential of various plant extracts against a number of plant pathogens has been reported^18,19^. Extracts of rhizomes, roots, leaves, stems, bulbs and other plant parts are used as seed treatments to control pathogens causing seed-borne diseases^20^. Owolade et al.^16^ reported significant inhibitory effects of aqueous extracts of *Ocimum gratissimum*, *Acalypha ciliata*, *Vernonia amyygdalina*, *Mangifera indica* and *Azadirachta indica* against *F. moniliforme*. However, very few reports have indicated the in vivo inhibitory effects of plant extracts against *F. verticillioides* under axenic and natural conditions in the field^21,22^*.*

Leaf extracts of the tree *Jacaranda mimosifolia*, one of 49 species in the *Jacaranda* genus, are known to have biological activities. Hexane and water extracts have been shown to inhibit the growth of *Escherichia coli*, ethanol extract to inhibit the growth of *Bacillus cereus*, and all three extracts to inhibit *Staphyloccus aureus*^23^. Our previous work showed that *J. minosifolia* leaf extract was an effective inhibitor of wheat leaf rust (*Puccinia triticina*) spore germination, and that it stimulated defense-related gene expression and the subsequent accumulation of PR proteins in the apoplast of inoculated wheat leaves^24^. Extracts from various species in the *Jacaranda* genus have been shown to contain specific triterpenes, various flavonoids and a range of acetosides^25^.

In our research program we became interested to determine whether *J. minosifolia* leaf extracts might have a protective effect against *F. verticillioides* when applied to maize seeds well in advance of pathogen inoculation of the stalk. The main aims of this study were: (1) to formulate seed treatments using *J. mimosifolia* leaf extracts with SA, mefenoxam and Tween-80 (as an emulsifier) and to determine their effectiveness in controlling *F. verticillioides*-mediated stalk rot of maize in both pot and field experiments; (2) to monitor changes in physiological and biochemical responses and in defense-related proteins in maize and identify possible defense mechanisms against stalk rot.

## Material and methods

### Plant seeds and pathogen

Seeds of maize cv. Islamabad Gold, which is susceptible to stalk rot, and the associated fungal pathogen, *Fusarium verticillioides*, were obtained from the National Agricultural Research Centre (NARC), Islamabad, Pakistan.

### Preparation of plant leaf extracts and determination of in vitro fungicide activity

Fully expanded leaves were collected from three *Jacaranda mimosifolia* trees growing in the Quaid-i-Azam University campus, Islamabad, and identified by the National Herbarium, Department of Plant Sciences, Quaid-i-Azam University, Islamabad. The leaves were washed thoroughly with distilled water and shade-dried at room temperature for 7–8 days. The dried leaves were ground uniformly using an electric grinder. The powdered plant material (250 g) was extracted with occasional shaking for either 4 days in 1 L 100% methanol or for 24 h in 1 L sterile distilled water^26^. The liquid phase of the settled extracts was then filtered through WHATMAN NO. 1 filter paper and the methanol filtrate evaporated to dryness using a rotary evaporator at room temperature (30 °C). The dried methanolic extract was stored in an air-tight container at 4 °C until further use. For both the dried methanolic extract and the water extract, concentrations of 0.8, 1.0 and 1.2% (w/v for the methanolic extract; v/v for the water extract) were used for a phytotoxicity assay under axenic conditions and in vitro antifungal assay, as described below. Reconstituted methanolic extracts were prepared by completely dissolving 8, 10 and 12 mg dried extract in 1 mL of dimethyl sulfoxide (DMSO, SIGMA-ALDRICH D2650, MO) and filtered (0.22 µm; Millipore). The aqueous *J. mimosifolia* extracts showed stimulatory effects on maize plant growth as compared to methanolic extracts; thus, the aqueous extract was selected for the pot experiment (Supplementary Tables 6 and 7, Supplementary Figs. 3 and 4).

The agar tube dilution method was used for the determination of antifungal activity of *J. mimosifolia* leaf extracts^27^. Culture medium was prepared by dissolving 6.5 g of Sabouraud dextrose agar (MERCK, Darmstadt, Germany) per 100 mL distilled water. Aliquots (10 mL) of the Sabouraud dextrose agar solution were dispensed in screw-capped tubes or cotton-plugged test tubes, autoclaved at 121 °C for 21 min, and allowed to cool at 50 °C. The solutions were then loaded with 67 μL of either reconstituted methanolic or water extract, each at 0.8, 1.0 and 1.2% (v/v). The tubes containing the media were then allowed to solidify in slanting position at room temperature. Three slants for each concentration of methanol and water extracts were prepared.

The tubes containing solidified media and *J. mimosifolia* extract were inoculated with a 4 mm-diameter piece of *F. verticillioides* inoculum taken from a 7 day-old culture. Slants without extract but containing 67 μL of Terbinafine hydrochloride at 1 mg/mL (SIGMA-ALDRICH, Co., *St*. *Louis*, *MO*) were used as positive controls. Slants without extract but containing 67 μL DMSO or sterile water were used as negative controls. The tubes were placed in an incubator (equipped with fluorescent lights) at 28 °C for 7 days with a photoperiod of 12 h. Cultures were examined twice weekly to observe fungal growth during incubation. Mean growth values were obtained after 7 days and converted to percentage inhibition of mycelial growth in relation to the control treatment by using the following formula: MGI(%) = ((dc − dt)/dc) × 100, where MGI is minimum growth inhibition, and dc and dt represent mycelial growth diameter in control and treated samples, respectively.

### Selection of fungicide for in vivo experiment

Three commercial fungicides—Ridomil Gold SL (45.3% mefenoxam; SYNGENTA, Willmington, DE), Dithane M-45 (80% WP mancozeb; DOW AGRO SCIENCES, Pakistan) and *Score* (25% EC difenoconazole; SYNGENTA, Sydney, Australia)—were tested for their efficacy to inhibit the growth of *F. verticilloides*. The in vitro antifungal activities of the selected fungicides, each at three concentrations of active ingredient (0.05, 0.1 and 0.2%), were compared using the agar tube dilution method described above. Based on the inhibition of *F. verticilloides* mycelial growth, we found the most effective fungicide was mefenoxam (see “Results” section), which was therefore selected to prepare the formulations for the in vivo experiments.

### Preparation of inoculum and seed treatments for pot and field experiments

An inoculum suspension in potato dextrose broth (PDB; 0.4% potato starch, 2% dextrose; BD Difco, Sparks, MD) from *F. verticillioides* culture was prepared following the method of Tesso et al.^28^ with some modifications. The suspension was incubated at room temperature on a shaker (EXCELLA E24, Eppendorf, NY) at 140 rpm with a 12 h photoperiod (under fluorescent lights) until microconidia were produced. Four layers of cheesecloth were used to strain and separate the mycelial mass from the microconidia. The concentration was adjusted to the required dose (1 × 10^6^ conidia mL^−1^) by diluting the suspension with sterile phosphate-buffered saline (PBS) solution.

Various seed treatments were prepared from the leaf extracts of *J. mimosifolia*, mefenoxam (as Ridomil Gold SL), SA (SIGMA-ALDRICH, Saint Louis, MO) and Tween-80 (0.6% v/v). All formulations were packaged in 1-L plastic bottles and stored in the dark at room temperature prior to application.

Based on in vitro tests on the effect of the methanolic and water extracts at the three concentrations (0.8, 1.0 and 1.2%) on *F. verticillioides* mycelial growth (see “Results” section), water extracts at 1.2% were chosen for the pot and field studies. Seeds of maize cv. Islamabad Gold were surface-sterilized for both the pot and field experiment with 95% (v/v) ethanol followed by shaking in 10% (v/v) Clorox bleach (5.25% sodium hypochlorite as the active ingredient diluted tenfold to a final concentration of 0.5%) for 2–3 min. The seeds were then rinsed thoroughly three times with sterile distilled water. Mefenoxam (0.2%), SA (4 mM) and *J. mimosifolia* water extract (1.2%) were applied as seed soaking treatments alone (as controls) and in combination (mixtures) for 1 h followed by air drying prior to sowing with three replicates for each treatment.

### Pot experiments

Pot experiments under glasshouse conditions were performed twice, in consecutive years. Seed treatments with *J. mimosifolia* extract and SA were evaluated for their effects on the growth and physiology of uninoculated maize plants compared to untreated controls (Supplementary Fig. 2). Different seed treatments comprising the *J. mimosifolia* extract, mefenoxam and SA, both separately and in combination (Table 1), were tested against *F. verticillioides*. A mixture of soil and sand at 3:1 (w/w), pH 7.0, with available nutrients Na, K, P, Mg and Ca at 21, 13, 11, 0.8 and 37 µg/g (dry weight), respectively, was added to sterilized plastic pots (25 cm diameter × 40 cm height). Ten seeds were sown in each of three pots per treatment and thinned to six plants per pot after germination. Plants were grown with an average temperature of 30 °C during the day (10–13 h) and 25 °C during the night. Placement of the pots in the glasshouse followed a completely randomized design.

**Table 1:** The 10 maize seed treatments developed and tested in pot and field experiments.

Treatments	Symbols
Uninfected control Infected with *F. verticillioides* Mefenoxam full dose (0.2%) + *F. verticillioides* *J. mimosifolia* full dose (1.2%) + *F. verticillioides* Salicylic acid full dose (4 mM) + *F. verticillioides* Mefenoxam half dose (0.1%) + *F. verticillioides* *J. mimosifolia* half dose (0.6%) + *F. verticillioides* Salicylic acid half dose (2 mM) + *F. verticillioides* *J. mimosifolia* (0.6%) + Mefenoxam (0.1%) + *F. verticillioides* *J. mimosifolia* (0.6%) + Salicylic acid (2 mM) + *F. verticillioides*	Control Fv Mef + Fv Jm + Fv SA + Fv ½ Mef + Fv ½ Jm + Fv ½ SA + Fv ½ (Jm + Mef) + Fv ½ (Jm + SA) + Fv

At the tasseling stage (60 days after sowing), plants that were to be inoculated with *F. verticillioides* were tagged with distinct tapes. Inoculation was performed by injecting 1 mL suspension (1 × 10^6^ conidia mL^−1^) using a syringe into the second node of the maize stem following the method of Tesso et al.^28^. The stem was covered with tape at the site of inoculation to provide humidity to assist fungal proliferation. Control plants were mock-inoculated with 1 mL of sterile PBS buffer.

### Field experiments

The field experiments were performed twice, in consecutive years, in a wire house at the Quaid-i-Azam University, Islamabad (Pakistan) to assess the efficacy of seed treatments against *Fusarium* stalk rot infection. The field soil was at pH 7.5 and contained the available nutrients Na, K, P, Mg and Ca at levels of 10.4, 12.2, 17.3, 5.7 and 15.9 µg/g (dry weight), respectively. The treated seeds (see above) were sown in field plots measuring 1 × 1 m^2^ comprised of two rows (45 cm apart) with five plants per row. Each of three replicate plots represented a single seed treatment and the plots were arranged according to a randomized complete block design. Pathogen inoculation was performed as described for the pot experiments.

### Sample collection and measurement of lesion length for pot and field experiments

Three randomly selected whole plants were harvested (during a 2-h period beginning at 10 am) from each of the three replicate pots or plots—one plant for each of three different stages of maize development. These stages were the silk, blister and dough stage corresponding to 7, 14 and 21 days after the inoculation with *F. verticillioides*, respectively. Leaves from these plants were used to determine the activities of defense-related enzymes, protein concentration and protein profile (by SDS-PAGE). For the study of the expression of PR and defense-related genes by RT-qPCR, one fully expanded leaf from a randomly selected plant was harvested from each of the three replicate pots or plots 72 h after inoculation.

At the dough stage (21 days after inoculation), three plants from each pot and five plants from each plot were harvested and scored for disease reduction. The disease rating—average lesion length per pot/plot—was determined by splitting the stalks longitudinally and measuring the length (in cm) of the visible necrotic lesion^28^.

### RNA extraction and RT-qPCR

RNA was extracted from leaves using TRIZOL reagent (THERMOFISHER SCIENTIFIC, Waltham, MA) according to the manufacturer’s instructions and quantified using a NanoDrop 1000 Spectrophotometer (THERMO SCIENTIFIC) at a 1:10 (v/v) dilution. Purified RNA was used as template to synthesize cDNA using a First Strand cDNA synthesis kit (THERMO SCIENTIFIC) as per the manufacturer’s instructions. Six genes known to be involved in plant responses to infection, as well as the housekeeping gene, *Actin*, used as the internal control, were selected for RT-qPCR analysis (primer sequences are given in Supplementary Table 1). Real-time polymerase chain reactions were performed using 12.5 μL of the 2 × Maxima SYBR Green/ROX qPCR Master Mix (THERMO SCIENTIFIC), 0.4 μL of forward primer (0.3 μM), 0.4 μL of reverse primer (0.3 μM), 2.0 μL of template (30 ng) and nuclease-free water to 25 μL. Reactions were performed in triplicate (technical replicates) for each sample , including negative controls in which cDNA was substituted by the same volume of water. The STEPONEPLUS REAL-TIME PCR System (APPLIED BIOSYSTEMS) was used with the following conditions: initial denaturation at 95 °C for 10 min, followed by 40 cycles of 95 °C for 15 s, 60 °C for 60 s and 72 °C for 20 s and extension at 72 °C for 1 min. The results were interpreted using delta ct values^29^.

### Determination of acid invertase (AI) activity

AI activity of leaves was assayed according to Hwang and Heitefuss^30^ with some modifications. Leaf segments (0.25 g) were excised after inoculation of the pathogen from control and infected leaf tissues, immersed in ice-cold ethyl acetate for 20 min and finally washed in ice-cold distilled water. Each sample was incubated in 0.1 M sodium phosphate buffer (pH 5.6), 0.5 M sucrose and distilled water in a water bath at 30 °C for 60 min. The remaining water on the leaf surface was blotted with filter paper. Each leaf sample was transferred into a 20-mL vial containing 2 mL of 0.5 M sucrose, 2 mL of 0.1 M sodium phosphate buffer (pH. 5.6) and 6 mL of double-distilled water. The vials were incubated in a shaking water bath at 30 °C for 60 min. AI activity was measured by reading the absorbance of each sample at 280 nm in a spectrophotometer (HITACHI Model: U-1100 573 × 415).

### Determination of protease activity

Protease activity was assessed by the method of McDonald and Chen^31^. Leaf tissue (100 mg) was incubated with 4 mL of the substrate (1% casein in 0.1 M sodium citrate buffer of pH 7.0) for 1 h at 30 °C. Five millilitres of 5% trichloroacetic acid (TCA) was added to precipitate the residual protein. The precipitate was allowed to settle for 30 min and the contents of the tubes was filtered through filter paper (WHATMAN No. 40). After filtration, a 1-mL aliquot of the filtrate was mixed with 5 mL of alkaline reagent mixture, which was prepared by mixing 100 mL of sodium carbonate (2% w/v), 1 mL of sodium potassium tartrate (2.7% w/v) and 1% (w/v) copper sulfate. Two millilitres of 1 M sodium hydroxide was then added to make the solution alkaline. After a minimum of 10 min, Folin-Ciocalteu-phenol reagent (0.5 mL) was added and the contents were mixed. The absorbance of the blue color produced in each sample was measured at 660 nm after 30 min using a spectrophotometer. One unit of protease activity was defined as the amount of enzyme required to produce an increase in optical density at 660 nm of 0.1 h^−1^ at 30 °C at pH 7.0.

### Estimation of polyphenol oxidase (PPO) activity

PPO activity was determined following the method of Kar and Mishra^32^ with some modifications. Leaf tissue (1 g) was homogenized in 2 mL of 0.1 M sodium phosphate buffer (pH 6.5) and centrifuged at 10,000×g for 25 min at 4 °C. The supernatant was used as an enzyme extract. The 3-mL reaction mixture contained 25 mM phosphate buffer (pH 6.8), 0.1 mM pyrogallol and 0.1 mL enzyme extract. The control reaction mixture contained no pyrogallol. The absorbance of each sample was recorded at 420 nm.

### Estimation of peroxidase activity (POD)

Leaf tissue (1 g) was homogenized in 5 mL of 0.05 M phosphate buffer (pH 7.0) containing 10% polyvinylpyrrolidone (SIGMA) and 0.1 M ethylene diaminetetraacetic acid (EDTA) (SIGMA), and centrifuged at 14,000 rpm for 20 min at 4 °C. The supernatant was used for the POD assay^33^. POD activity was measured by the method of Vetter et al.^34^ as modified by Gorin and Heidema^35^. The assay mixture contained 0.1 mL enzyme extract, 1.35 mL 0.1 M mM MES buffer (pH 5.5), 0.05% H_2_O_2_ and 0.1% phenylenediamine. Changes in the absorbance in each sample were recorded at 485 nm for 3 min.

### Estimation of chitinase activity

Chitinase activity was determined by colorimetric assay using the purple dye-labeled biopolymeric substrate, CM chitin-RBV (LOEWE BIOCHEMICAL, Germany)^36^. CM-chitin-RBV (200 µL of 2 mg/mL) was mixed with 300 µL of leaf protein extract and 300 µL of 10 mM Tris–HCl, pH 7.5, containing 1% Triton X-100. The mixture was incubated at 37 °C for 3 h. The reaction was stopped by the addition of 200 µL of 2 M HCl. Samples were cooled on ice for 15 min and then centrifuged at 20,000 × *g* for 10 min to remove the non-degraded substrate. The supernatant was collected and the absorbance measured at 550 nm. One unit of chitinase activity represented an increase of absorbance of 0.1 at 550 nm^37,38^.

### Estimation of phenylalanine ammonia lyase (PAL) activity

The PAL enzyme was extracted and partially purified by the method of Suzuki et al.^39^. Fresh leaves (1 g) were ground at 4 °C in 5 mL of 0.1 M sodium borate buffer (pH 8.8). Homogenates were centrifuged at 12,000 × *g* for 15 min at 4 °C and the supernatant was used as the enzyme extract. Reaction mixtures consisting of 500 μL sodium borate buffer (pH 8.7) and 250 μL enzyme extracts were pre-incubated for 5 min at 40 °C. The reaction was started by the addition of 300 μL of 50 mM l-phenylalanine (SIGMA-ALDRICH) and, after incubation for 1 h at 40 °C, stopped by adding 50 μL of 5 N HCl. The reaction mixture was centrifuged again (12,000 × *g* for 15 min) prior to injection into an HPLC (SHIMADZU, C-R4A Chromatopac; SCL-6B system controller) featuring a ZORBAX SB-C18 analytical column (4.6 × 150 mm, 5 μm particle size, AGILENT, Germany) and a U.V. detector at room temperature. The mobile phase consisted of 57% acetonitrile in water with a flow rate of 0.5 mL min^−1^. Detection of *trans*-cinnamic acid (*t*-CA) was based on retention time and performed at 275 nm. The activity of PAL was expressed as nmol *t*-CA min^−1^ g^−1^ of fresh mass in relation to the peak area of a *t*-CA standard solution (1 mg/100 mL sodium borate buffer, pH 8.7).

### Estimation of protein content

Protein content of the leaves was determined following the method of Lowry et al.^40^ with bovine serum albumin (BSA) as standard. Fresh leaves (0.1 g) were ground in 1 mL of phosphate buffer pH 7.5 with a mortar and pestle and the homogenate centrifuged at 3,000 rpm for 10 min at room temperature. The supernatant was transferred to test tubes and distilled water added to make a total volume of 1 mL. Alkaline copper sulfate reagent (1 mL) was added and, after shaking for 10 min, 0.1 mL of the Folin’s reagent was added and the mixture incubated for 30 min. The absorbance was recorded at 650 nm against a blank (1.0 mL of 0.5 M sodium hydroxide).

### Protein profiling by SDS-PAGE

Leaf proteins were precipitated by trichloroacetic acid (TCA)/acetone and purified by phenol-based extraction following the method of Wang et al.^41^ with some modifications. Leaf tissue (1 g) was homogenized in 4 mL of 10% (w/v) TCA/acetone and the homogenate was centrifuged at 12,000 g at 4 °C for 5 min. The supernatant was precipitated overnight at − 20 °C and pelleted by centrifugation at 5000g for 10 min. The supernatant was discarded and the soluble protein pellet was re-suspended in 0.07% 2-mercaptoethanol (v/v) in cold acetone. The samples were stored at − 20 °C for 1 h, and then centrifuged at 10,000×*g* for 15 min at 4 °C; this step was repeated twice. The supernatant was discarded and pellet was dried under vacuum for ~ 1 h and stored at -80 °C.

The pellets were dissolved in 10% glycerol, 2.3% SDS, 5% 2-mercaptoethanol, 0.25% bromophenol blue, 63 mM Tris–HCl (pH 6.8) and heated at 70 °C for 10 min. Polypeptides in a sample containing equal volumes (10 μl) of protein extract were separated by SDS-PAGE^42^. Acryl and Bis-acryl amide gradient gels (pH 7) (4–12%) were used and proteins were resolved under 200 V (constant) in a Protean II system (BIO-RAD) until the sample buffer containing bromophenol blue reached the bottom of the gel (40–50 min). Protein bands were visualized by staining with a solution containing 0.1% (w/v) Coomassie R250, 40% ethanol and 10% acetic acid, and a destaining solution containing 10% (v/v) ethanol and 7.5% (v/v) acetic acid. Gels were analyzed using a 2000 Quantity-One 4.5.2 gel documentation system (BIO-RAD, USA) and band density was further analyzed by IMAGEJ^43^.

### Statistical analysis

The pot and field experiment data were subjected to a generalized linear mixed model (GLMM) using ORIGIN PRO 2016 (ORIGINLAB, Northampton, MA) (Supplementary Tables 2 and 3) and statistical analysis using STATISTIX version 8.1. Analyses of the data generated from the repeated pot and field experiments (2010 and 2011) were performed to determine the seasonal effect. Comparisons among mean values of treatments were made by Least Significant Difference (LSD) at *p* < 0.05^44^. Correlation among different factors was assessed by the linear regression/Pearson correlation coefficient test using ORIGINPRO 2016 (ORIGINLAB, Northampton, MA).

## Results

### In vitro antifungal potential of *J. mimosifolia* extracts and synthetic fungicides against *F. verticillioides*

Both the methanolic and aqueous leaf extracts of *J. mimosifolia*, at concentrations of 0.8, 1.0 and 1.2%, inhibited the mycelial growth of *F. verticillioides* (Table 2). The maximum inhibition was 96 and 97% with the 1.2% methanolic and aqueous extracts, respectively.

**Table 2:** Antifungal activity of *Jacaranda mimosifolia* extracts against *Fusarium verticillioides*.

Treatment	*Fusarium verticillioides* mycelial growth (cm)			
	Methanol extract			
	0.8%	1.0%	1.2%	
*Jacaranda mimosifolia*	0.67^d^ ± 0.05	0.57^b^ ± 0.05	0.33^b^ ± 0.05	
Positive control	0.77^c^ ± 0.12	0.77^b^ ± 0.12	0.77^b^ ± 0.12	
Negative control	9.8^b^ ± 0.05	10.00^a^ ± 0.05	9.80^a^ ± 0.03	
	Aqueous extract			
*Jacaranda mimosifolia*	0.73^ cd^ ± 0.05	0.60^b^ ± 0.1	0.43^b^ ± 0.05	
Positive control	0.83^c^ ± 0.05	0.83^b^ ± 0.05	0.83^b^ ± 0.05	
Negative control	10.00^a^ ± 0.08	10.00^a^ ± 0.05	10.00^a^ ± 0.05	

Data represent mean ± standard deviation (SD) of three replicates**.** All mean values in the same column with different letters are significantly different from each other (P < 0.05). Positive control: Terbinafine hydrochloride. Negative control for methanolic extracts: DMSO. Negative control for aqueous extracts: sterile distilled water.

Three selected synthetic fungicides—Ridomil Gold SL (mefenoxam), Dithane M-45 (mancozeb) and *Score* (difenoconazole), each at three concentrations—inhibited the mycelial growth of *F. verticillioides* (Table 3). At the highest concentration (0.2%), mefenoxam gave the maximum inhibition (79%), followed by difenoconazole (71%) and mancozeb (61%).

**Table 3:** Antifungal activity of synthetic fungicides against *Fusarium verticillioides.*

Treatment	*Fusarium verticillioides* mycelial growth (cm)		
	0.05%	0.10%	0.20%
Mefenoxam	3.20^b^ ± 0.71	2.80^c^ ± 0.54	2.10^c^ ± 0.54
Mancozeb	5.30^a^ ± 0.92	4.93^a^ ± 0.81	3.90^a^ ± 0.57
Difenoconazole	4.87^a^ ± 0.76	3.43^b^ ± 0.69	2.93^b^ ± 0.61
Negative control	10.00 ± 0.71	9.80 ± 0.69	10.00 ± 0.62

Percentage values are concentrations of the fungicides tested. Data represent mean ± standard deviation (SD) of three replicates**.** All mean values in the same column with different letters are significantly different from each other (P < 0.05). Negative control: sterile distilled water.

### Effects of seed treatments with *J. mimosifolia* extracts on leaves of maize plants with stems inoculated with *F. verticillioides*: pot and field experiments

Values for all the physiological and biochemical parameters measured, including defense-related enzyme activities, for both pot and field experiments, were found to increase at 14 days (blister stage) and thereafter decline at 21 days (dough stage) after stem inoculation with *F. verticillioides*.

**Figure 1:**
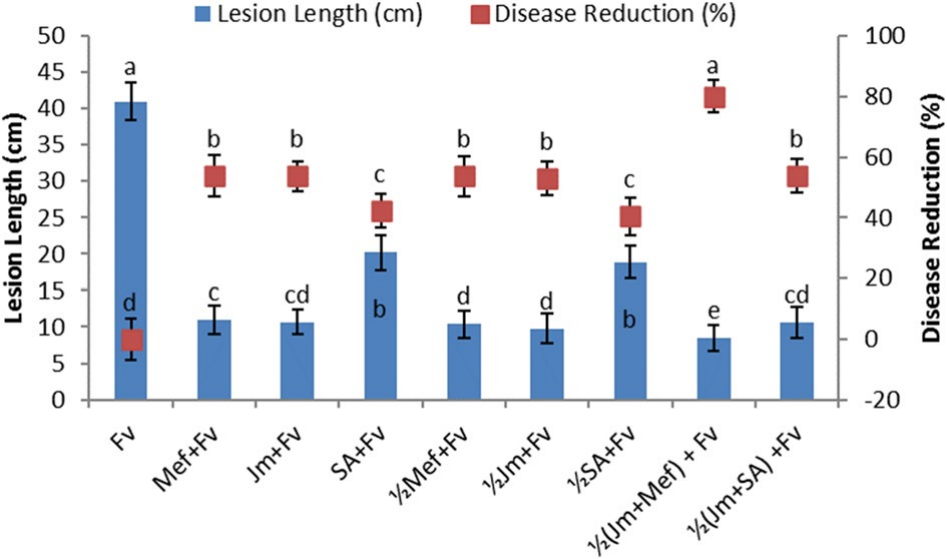
Effect of seed treatments with *J. mimosifolia* extract and control formulations on stem lesion length and disease reduction in maize inoculated with *F. verticillioides* (21 days after inoculation) in pot experiments under controlled conditions. Data are expressed as mean ± standard deviation (SD) of three replicates. Mean values with different letters are significantly different from each other according to the least significant difference (*LSD*) test at P < 0.05. See Table 1 for abbreviations.

**Figure 2:**
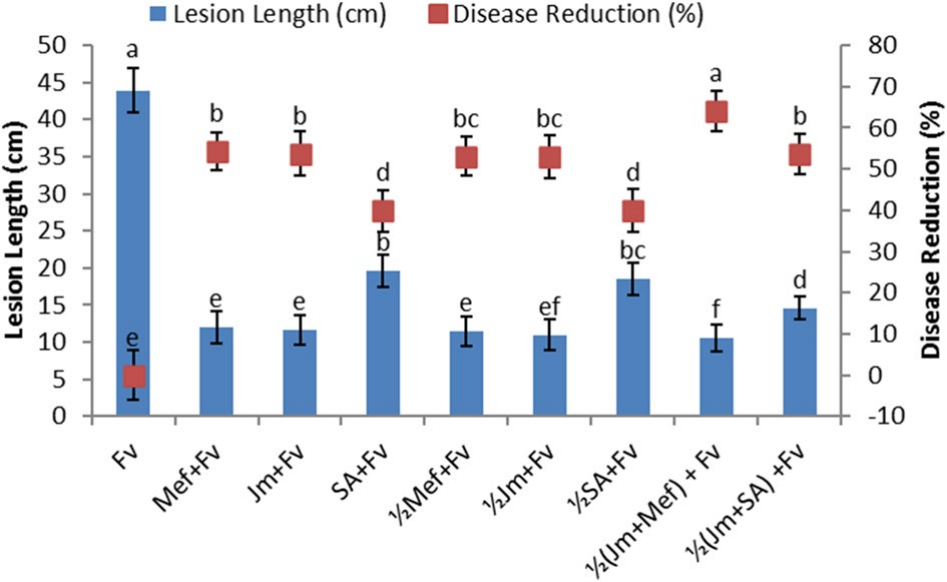
Effect of seed treatments with *J. mimosifolia* extract and control formulations on stem lesion length and disease reduction in maize inoculated with *F. verticillioides* (21 days after inoculation) in field experiments. Data are expressed as mean ± standard deviation (SD) of three replicates. Mean values with different letters are significantly different from each other according to the least significant difference (*LSD*) test at P < 0.05. See Table 1 for abbreviations.

In the pot experiment, full as well as half-dose applications of aqueous leaf extract of *J. mimosifolia*, SA and mefenoxam, each separately, resulted in a significant disease reduction in maize stalk rot ranging from 33 to 54% compared to the infected control (Fig. 1). Full-dose applications of *J. mimosifolia* leaf extract, SA, as well as mefenoxam significantly decreased the lesion length by 73, 74 and 51%, respectively, compared with the infected control, while the half-dose applications decreased the lesion length by 75, 76 and 54%, respectively (Fig. 1). In the combined applications, *J. mimosifolia* extract enhanced the effect of the SA and mefenoxam, reducing the lesion length by 79 and 74%, respectively, and giving a disease reduction of 80 and 54%, respectively, compared to the infected control. Among all the treatments, the maximum disease reduction (80%) was achieved using the half-dose application of *J. mimosifolia* with half-strength mefenoxam. In the field experiment, *J. mimosifolia* extract enhanced the effect of the mefenoxam, reducing lesion length by 76% and giving maximum (64%) disease reduction compared to the untreated infected control (Fig. 2).

### Differential expression of defense/pathogen-related genes at the transcript level

Expression patterns of defense-related genes in the leaves following seed treatments with *J. mimosifolia* extracts and stem inoculation with *F. verticilloides* in both pot and field experiments were analyzed using RT-qPCR (Fig. 2, Supplementary Tables 4 and 5. All the treatments resulted in substantial up-regulation of the selected genes in response to *F. verticilloides* inoculation, which correlated significantly with the reduction in lesion length.

### Expression of genes for chitinase, glucanase and lipid-transfer protein

**Figure 3:**
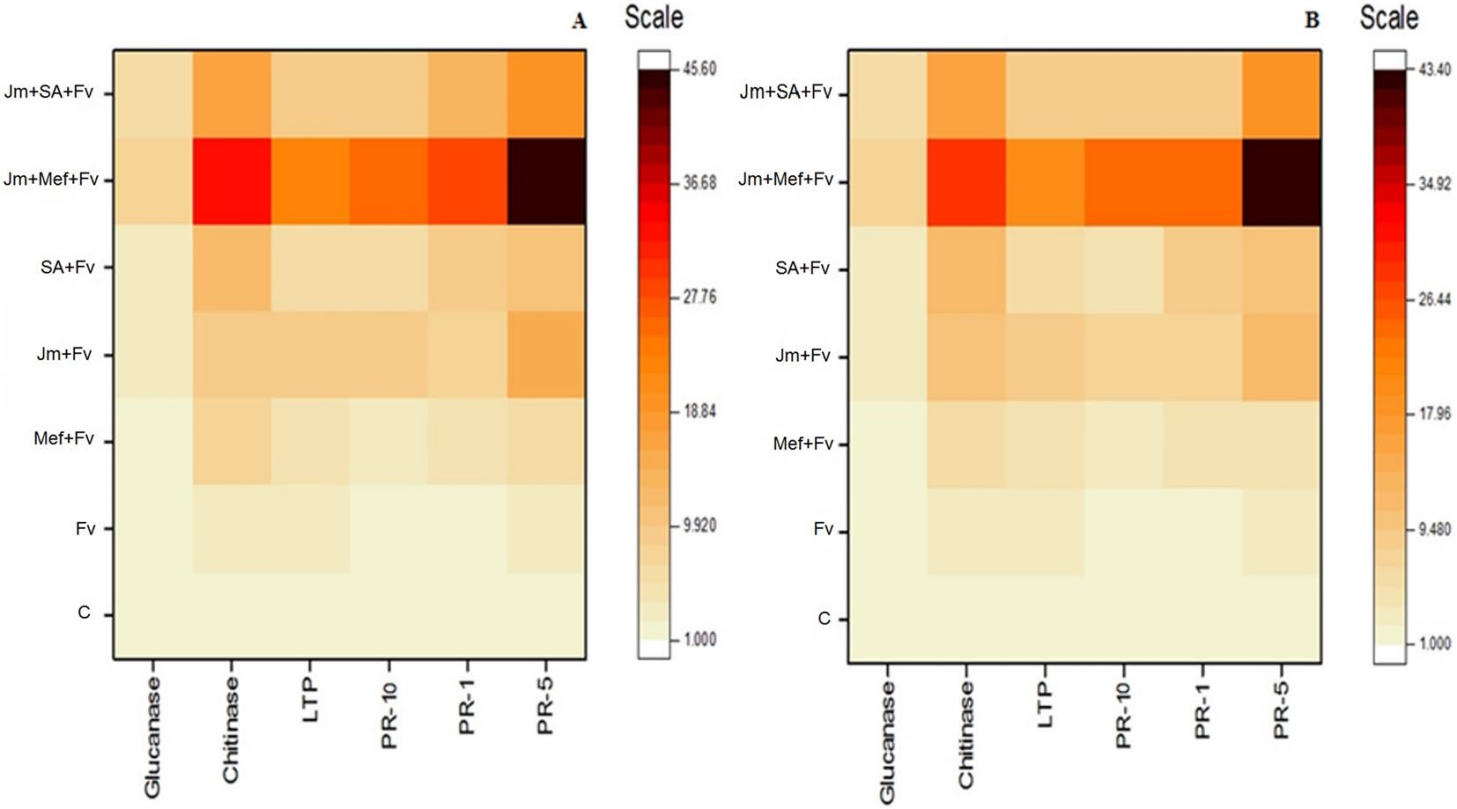
Heat maps showing the effect of seed treatments with *J. mimosifolia* and control formulations on relative expression profiles in maize leaves of selected defense-related genes 72 h after inoculation of stems with *F. verticillioides* in (**A**) pot experiment (**B**) field experiment. Relative transcript abundance was determined using RT-qPCR. Values given with color scale represent fold up-regulation. Data represent means of 2 years’ pooled experiments with three replicates for both pot and field experiments. Data are expressed as mean ± standard error of three replicates shown in Supplementary Tables 4 and 5. See Table 1 for abbreviations.

In response to various seed treatments with *J. mimosifolia* extracts, the expression in leaves of chitinase, glucanase and lipid-transfer protein (LTP) genes was up-regulated in both pot and field experiments 72 h after stem inoculation. Full-dose seed treatments with *J. mimosifolia* extract up-regulated the expression of chitinase, glucanase and LTP by 6, 2 and sixfold in the pot experiment and by 7, 2 and sixfold in the field experiment, respectively, compared to the inoculated control. The seed treatment with half-dose *J. mimosifolia* extract in combination with half-strength mefenoxam significantly up-regulated the expression of chitinase, glucanase and LTP by 30, 7 and 20-fold in the pot experiment and by 25, 6 and 18-fold in the field experiment, respectively, compared to the untreated inoculated control. All other treatments induced increases in the level of expression of chitinase, glucanase and LTP genes when compared to the untreated, inoculated control in both pot and field experiments (Fig. 3A,B, Supplementary Tables 4 and 5). Overall, the increase in the level of expression of the selected genes was higher in the pot experiment than in the field.

### Expression of genes encoding pathogenesis-related proteins (*PR-1, PR-5* and *PR-10*)

Seed treatment with *J. mimosifolia* extracts alone as well as in combination with half-strength mefenoxam and SA upregulated the expression in leaves of *PR-1, PR-5* and *PR-10* genes after stem inoculation with *F. verticilloides* in both pot and field experiments. All treatments increased the level of expression of *PR-1* (3 to 24-fold in both the pot and field), *PR-5* (3 to 43-fold in the pot and 2 to 40-fold in the field) and *PR-10* (2 to 24-fold in the pot and 1 to 23-fold in the field) when compared to the inoculated control. A full-dose seed treatment of *J. mimosifolia* extract upregulated *PR-1, PR-5* and *PR-10* genes in the pot and in the field compared to the infected control. However, the maximum upregulation of the *PR-1, PR-5* and *PR-10* genes was observed with the combined application of half-dose *J. mimosifolia* with half-strength mefenoxam: 24, 43 and 24-fold, respectively, in the pot, and 24, 40 and 23-fold, respectively, in the field (Fig. 3A,B, Supplementary Tables 4 and 5).

### Effect of *J. mimosifolia* formulations on protein content and protein profile

**Figure 4:**
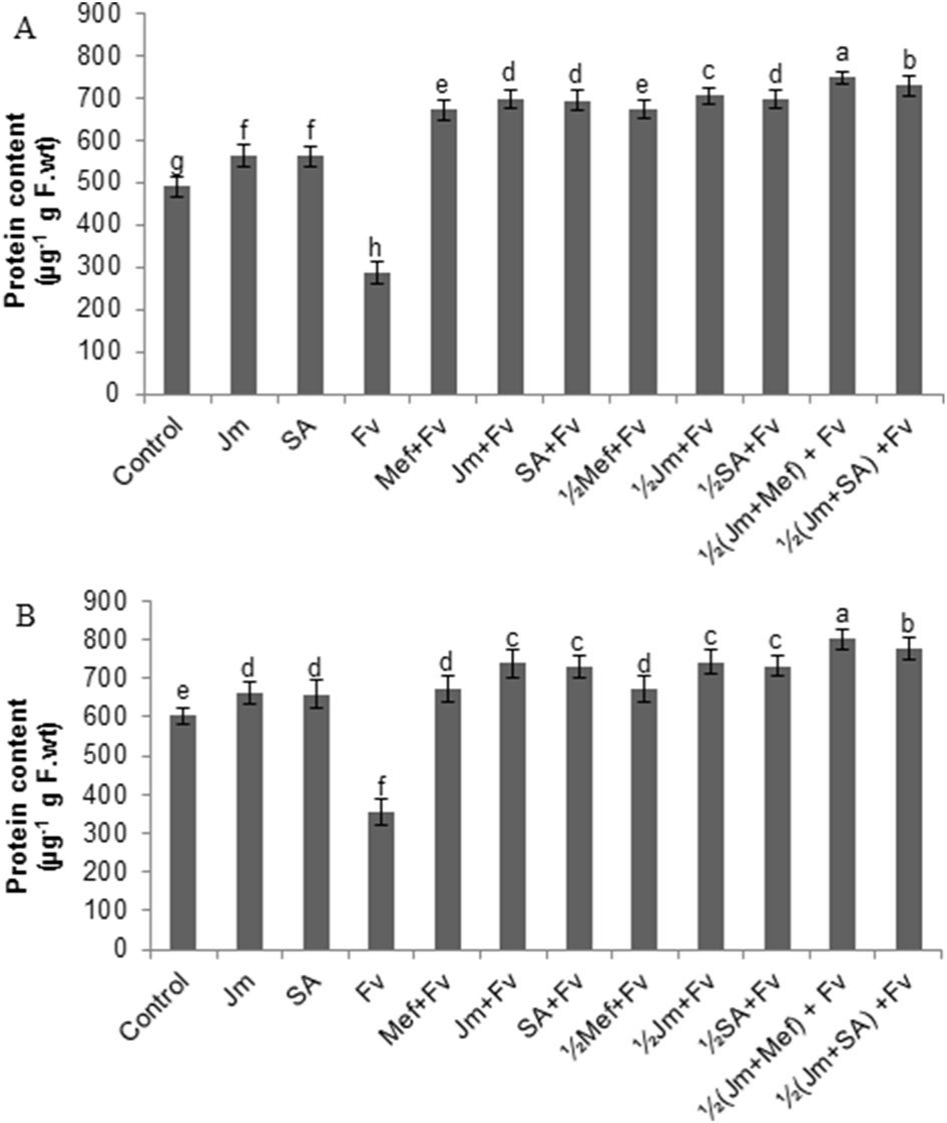
Effect of seed treatments with *J. mimosifolia* extract and control formulations on total soluble protein content of maize leaves 21 days after inoculation of stems with *F. verticillioides*. (**A**) Pot experiment, (**B**) Field experiment. Data represent means of 2 years’ pooled experiments for both pot and field experiments. Data are expressed as mean ± standard deviation (SD) of three replicates. Mean values with different letters are significantly different from each other according to the least significant difference (*LSD*) at P < 0.05. See Table 1 for abbreviations.

In both pot and field experiments, a single application of *J. mimosifolia* leaf extract and SA on uninoculated (uninfected) maize plants resulted in an increase in protein content by 14–15% and 9%, respectively. In both pot and field experiments, stem inoculation of *F. verticillioides* increased the total soluble protein content of the leaves for the treated plants compared to the untreated control plants (Fig. 3A,B). In the pot, *F. verticillioides* infection significantly decreased (by 71%) the total soluble protein content compared to the healthy control. Both full-dose and half-dose applications of mefenoxam, *J. mimosifolia* extract and SA to the seeds significantly increased the leaf protein content compared to the infected control (Fig. 4A). The maximum increase of 161% in protein content was given by the half-dose application of *J. mimosifolia* in combination with half-strength mefenoxam. In the field experiment, the *F. verticillioides* seed treatment gave a significant decrease (70%) in protein content as compared to the uninfected control (Fig. 4B). Both full-dose and half-dose applications of mefenoxam, *J. mimosifolia* extract and SA significantly increased protein content compared to the inoculated control. The combined half-dose application of *J. mimosifolia* with half-strength mefenoxam gave the maximum increase of 126% in leaf protein content.

**Figure 5:**
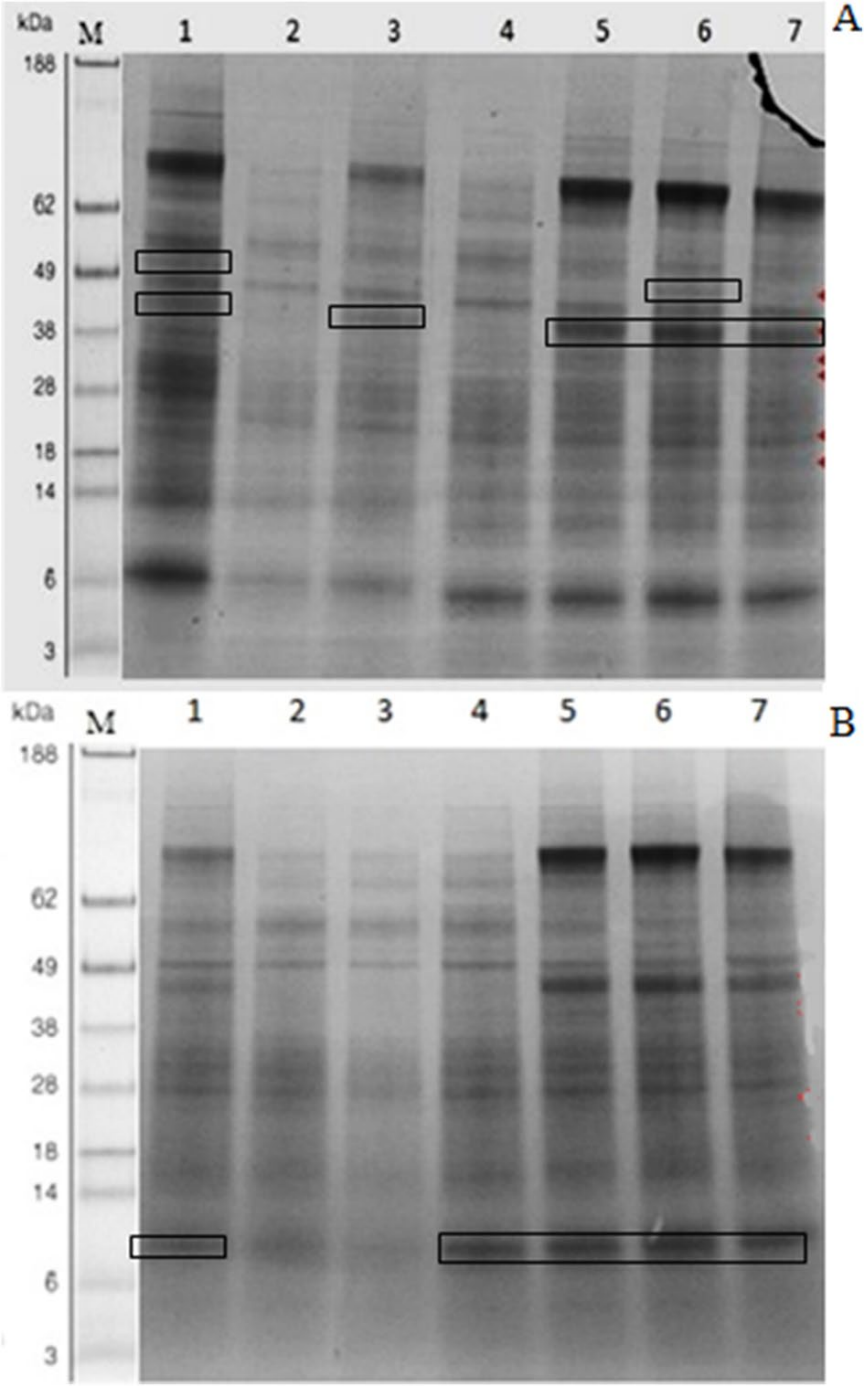
Effect of seed treatments with *J. mimosifolia* extract and control formulations on SDS-PAGE protein profiles of maize leaves from plants with stems infected with *F. verticillioides*. (**A**) Pot experiment, (**B**) Field experiment. Protein profile of maize leaves from 2nd year of experiment for both pot and field experiments. Encircled lanes indicate the induced protein bands. (**A**) Lane 1 = ½ (Jm + Mef) + Fv, Lane 2 = Fv, Lane 3 = Control, Lane 4 = Mef + Fv, Lane 5 = SA + Fv, Lane 6 = Jm + Fv, Lane 7 = ½ (Jm + SA) + Fv. (**B**) Lane 1 = Jm + Fv, Lane 2 = Control, Lane 3 = Fv, Lane 4 = Mef + Fv, Lane 5 = ½ (Jm + SA) + Fv, Lane 6 = ½ (Jm + Mef) + Fv, Lane 7 = SA + Fv. See Table 1 for abbreviations.

Leaf extracts were subjected to SDS-PAGE (Fig. 5) for the separation of newly induced proteins in response to pathogen inoculation. For the pot experiment, protein bands appeared in response to different treatments in which inoculation with *F. verticillioides* was compared with the untreated inoculated control sample (lane 2, Fig. 5A). A total of 21 bands were observed in maize given various seed treatments and inoculated with *F. verticillioides*, six more bands than found for the inoculated control. The maximum number of newly induced protein bands was recorded for the half-dose applications of *J. mimosifolia* with half-strength mefenoxam or SA. Single as well as combined half-dose applications of *J. mimosifolia* with mefenoxam induced a new protein band of 53 kDa (absent in the inoculated control). A new protein band of 48 kDa was induced by the single and combined applications of *J. mimosifolia* with mefenoxam and SA, which was absent in the infected control as well as in mefenoxam-treated plants.

In the field experiment, 20 polypeptide bands were induced in response to different treatments that included inoculation with *F. verticillioides* compared with the inoculated control (lane 3, Fig. 5B). Single and combined seed treatments with *J. mimosifolia* extract and SA induced new protein bands of 53 kDa and 48 kDa (absent in control and mefenoxam-treated plants). New polypeptide bands of 38 and 37 kDa were induced by all the seed treatments except infected and healthy control plants. Almost the same pattern of newly induced protein bands was observed in both pot and field experiments, indicating the highly induced defense enzymes after pathogen inoculation in response to seed treatments with plant extract.

A positive correlation was found between total soluble protein and protein band density values from SDS-PAGE of the leaf extracts. Correlation coefficients (R^2^) of 0.636 and 0.696 for total soluble protein and SDS-PAGE band density values were found for the pot and field experiment, respectively (Supplementary Fig. 1).

### Effect of seed treatment with *J. mimosifolia* extracts on the activity of defense-related enzyme and pathogenesis-related proteins in maize leaves

**Figure 6:**
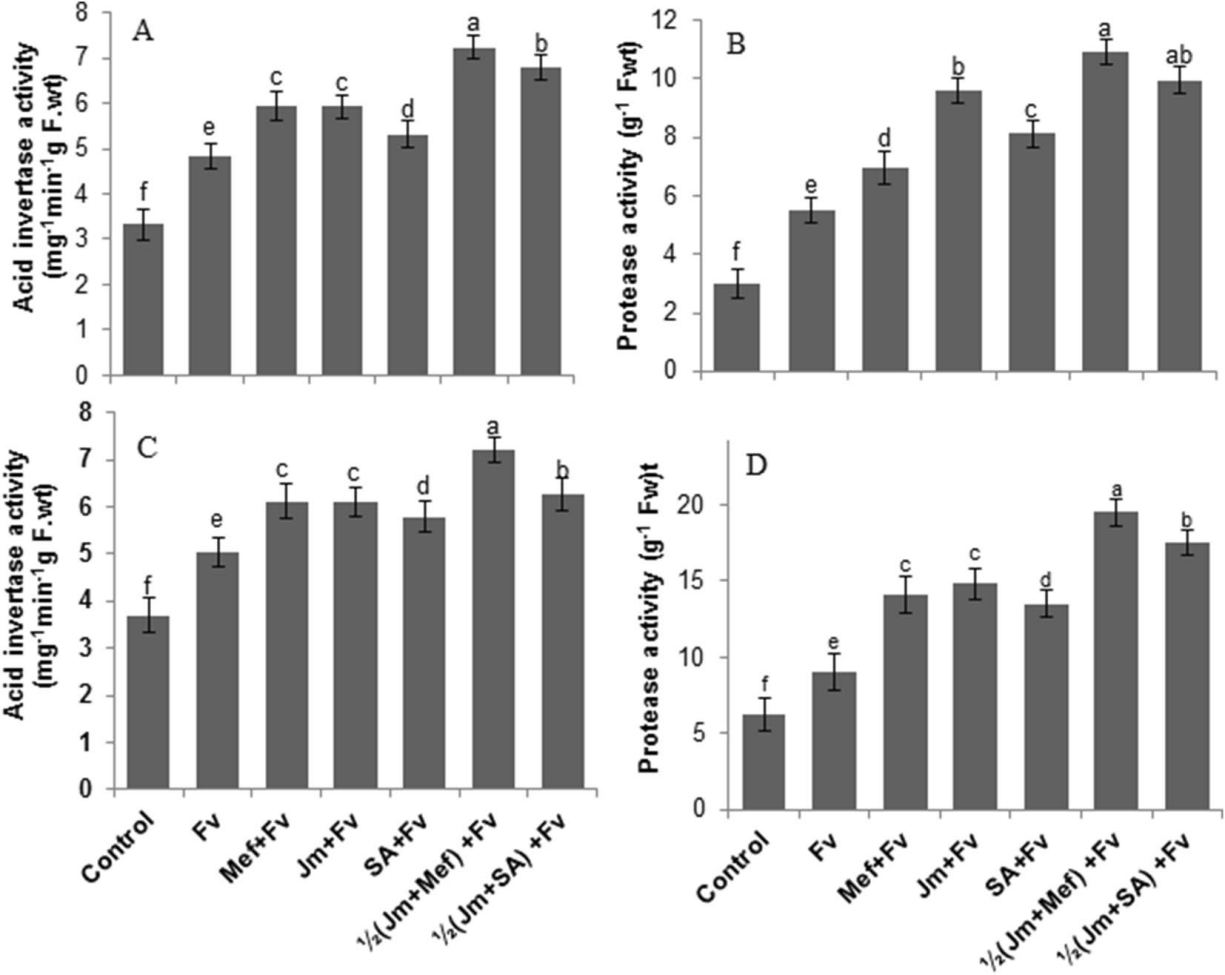
Effect of seed treatments with *J. mimosifolia* extract and control formulations on AI and protease activities of maize leaves from plants inoculated with *F. verticillioides* (21 d after inoculation). (**A**) AI activity (pot experiment), (**B**) Protease activity (pot experiment), (**C**) AI activity (field experiment), (**D**) Protease activity (field experiment). Data expressed as ± standard deviation of mean (SD) of three replicates. Mean values with different letters are significantly different from each other according to the least significant difference (*LSD*) at P < 0.05. See Table 1 for abbreviations.

Inoculation of *F. verticillioides* stimulated activities of all the defense-related enzymes tested in all treatments compared to the uninoculated control. In the pot experiment, acid invertase (AI) and protease activities in the leaves were higher in inoculated plants compared with the untreated plants (Fig. 6A,B). The mefenoxam seed treatment significantly increased the AI and protease activity by 22% and 64%, respectively, compared with the infected control. Full-dose seed treatment with *J. mimosifolia* extract increased the AI and protease activity by 22% and 75%, respectively, as compared to the infected control. The maximum increase in AI and protease activity (40% and 82%) was given by the half-dose *J. mimosifolia* extract combined with half-strength mefenoxam. In the field experiment, *F. verticillioides* we observed a significant increase in AI and protease activity by 36% and 46%, respectively, compared to the control (Fig. 6C,D). The combined treatment of half-dose *J. mimosifolia* with half-strength mefenoxam lead to the maximum increase of 41% and 116% in AI and protease activity, respectively.

**Figure 7:**
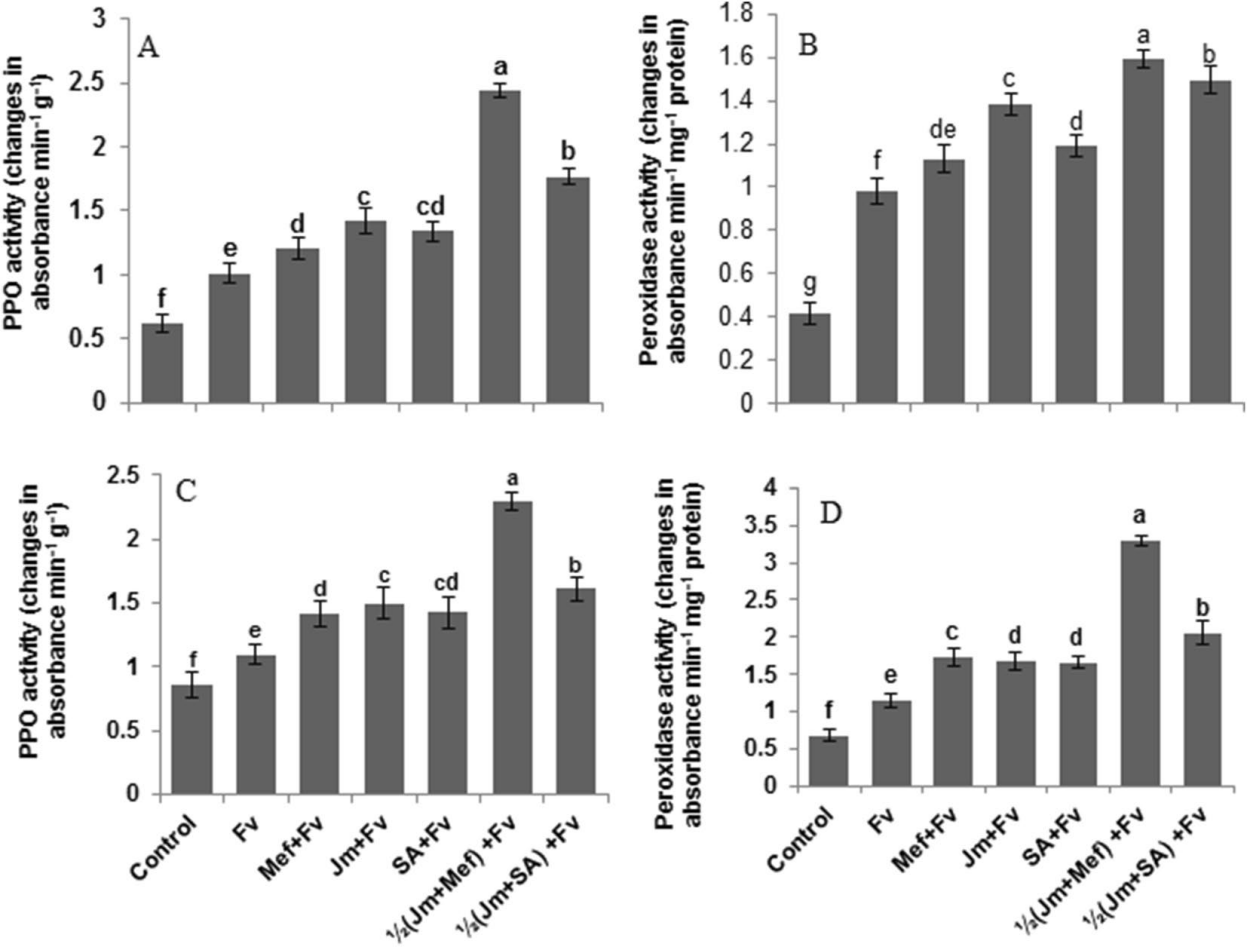
Effect of seed treatments with *J. mimosifolia* extract and control formulations on PPO and peroxidase activities of maize leaves from plants inoculated with *F. verticillioides* (21 d after inoculation). (**A**) PPO activity (pot experiment), (**B**) Peroxidase activity (pot experiment), (**C**) PPO activity (field experiment), (**D**) Peroxidase activity (field experiment). Data are expressed as ± standard deviation of mean (SD) of three replicates. Mean values with different letters are significantly different from each other according to the least significant difference (*LSD*) at P < 0.05. See Table 1 for abbreviations.

In both pot and field experiments, inoculation of *F. verticillioides* induced higher PPO and POD activity in the seed treated plants than in untreated controls (Fig. 7). In the pot, the mefenoxam seed treatment significantly increased the leaf PPO and POD activities by 29% and 40%, respectively, compared with the infected control (Fig. 7A,B). Thus the fungicide treatment had a stimulatory effect on these enzyme activities over and above the effect of the pathogen. The maximum increase of 98% and 62% in PPO and POD activity, respectively, was given by the combined treatment of *J. mimosifolia* with mefenoxam. In the field, *F. verticillioides* showed a significant increase (27% and 69%) in PPO and POD activity, respectively as compared to the infected control (Fig. 7C,D). The mefenoxam treatment significantly increased the PPO and POD activity by 29 and 40%, respectively. The combined application of half-dose *J. mimosifolia* extract with half-strength mefenoxam exhibited a maximum increase of 110 and 79% in PPO and POD activity, respectively.

**Figure 8:**
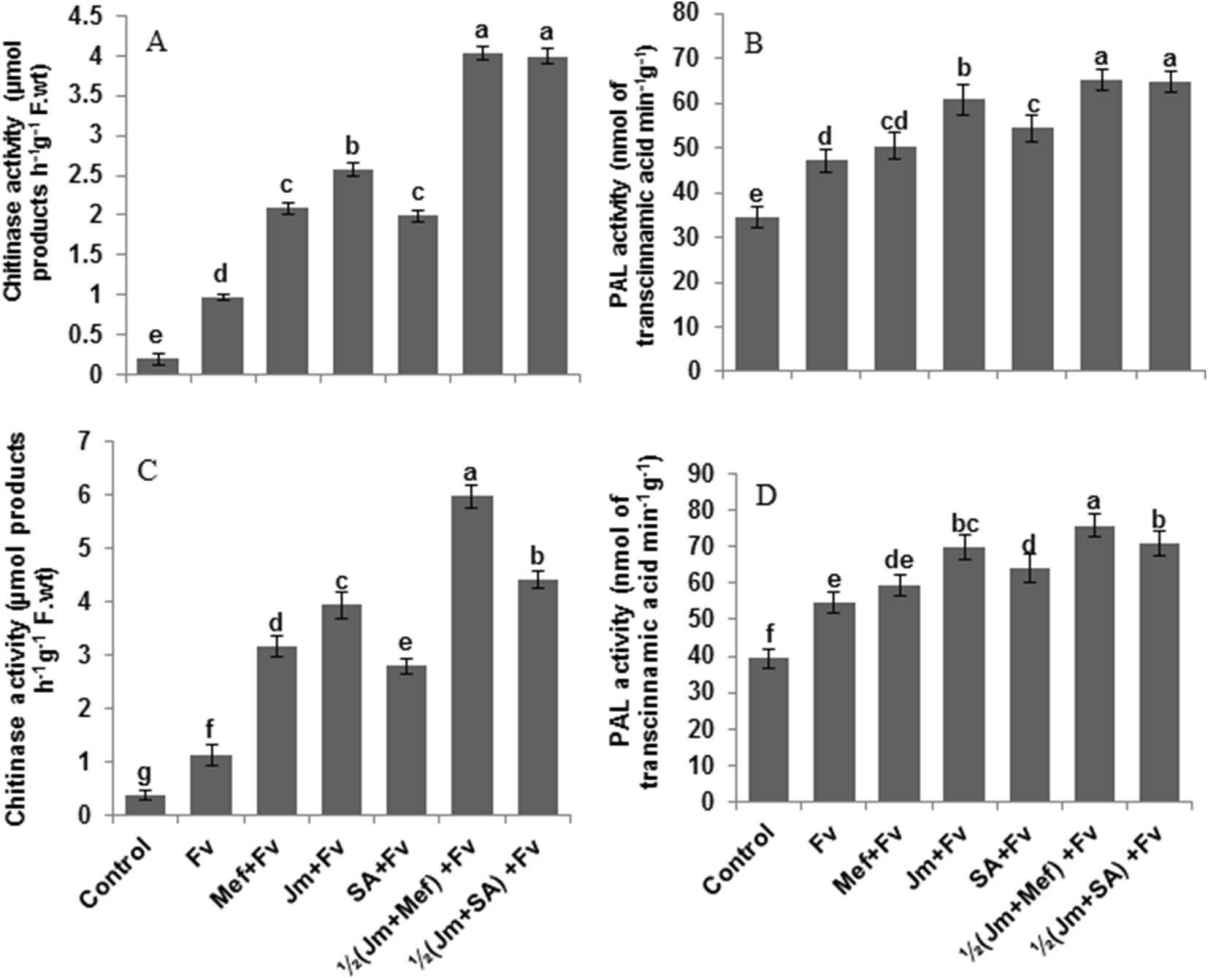
Effect of seed treatments with *J. mimosifolia* extract and control formulations on chitinase and PAL activities of maize leaves from plants inoculated with *F. verticillioides* (21 d after inoculation). (**A**) Chitinase activity (pot experiment), (**B**) PAL activity (pot experiment), (**C**) Chitinase activity (field experiment), (**D**) PAL activity (field experiment). Data are expressed as ± standard deviation of mean (SD) of three replicates. All means sharing the same letter are non-significantly different according to the least significant difference (*LSD*) at P < 0.05. See Table 1 for abbreviations.

In the pot, infection of maize plants with *F. verticillioides* was associated with a 395% and 37% increase in chitinase and PAL enzyme activity, respectively, as compared to the infected control (Fig. 8A,B). The maximum increase of 313% and 38% in chitinase and PAL activity, respectively, was exhibited by the combined half-dose application of *J. mimosifolia* extract with half-strength mefenoxam. In the field, *F. verticillioides* infection exhibited a significant increase in chitinase and PAL activity by 190% and 38%, respectively, as compared to the infected control (Fig. 8C,D). The maximum increase of 296% and 37% in chitinase and PAL activity, respectively, was given by the combined half-dose application of *J. mimosifolia* with mefenoxam.

## Discussion

Stalk rot caused by the fungus *Fusarium verticillioides* is prevalent worldwide in cereal-cultivating regions^45^. In this study, we examined the potential of *Jacaranda mimosifolia* leaf extracts as seed treatments to protect maize against *F. verticillioides.* Akinbode et al.^46^ had demonstrated the in vitro inhibitory effects of leaf extracts of *Tithonia diversifolia* (Mexican sunflower) against the mycelial growth of *F. verticillioides.* In our study, seed treatment with *J. mimosifolia* extracts was found to be an effective way to control stalk rot disease while reducing the required dose of synthetic fungicide.

We first observed that *J. mimosifolia* extract effectively inhibited the mycelial growth of *F. verticillioides *in vitro using Terbinafine as a standard fungicide positive control (Table 2). Terbinafine is active in vitro against a wide range of pathogenic fungi^47,48^.

The in vitro antifungal effects of the three selected fungicides on mycelial growth were investigated. Based on the inhibition of mycelial growth, the most effective fungicide was mefenoxam, which gave more than 70% inhibition against *F. verticilloides*; therefore it was selected for in vivo experiments. Mefenoxam in combination with fludioxonil, azoxystrobin and thiabendazole is reported to have inhibitory effects against *F. verticilloides*^49^. ApronMaxx RFC (mefenoxam 3.46% + fludioxonil 2.31%), CruiserMaxx Advanced (mefenoxam 3.21% + fludioxonil 1.07% + thiamethoxam 21.5%) and Warden RTA (mefenoxam 2.21% + fludioxonil 0.72%) have been reported to control *Fusarium* spp.^50^. Ramusi et al.^51^ have also reported mefenoxam (240 and 350 g/L) to control *Fusarium solani* in cowpea*.* We observed that seed treatment with *J. mimosifolia* extract augmented the effectiveness of the mefenoxam, as the half-dose formulation with half-strength mefenoxam gave significantly higher levels of inhibition of stalk rot infection than either the full-dose plant extract or full-strength mefenoxam applied alone. The efficacy of our treatments in partially protecting maize plants from stalk rot was surprising given the very long period (60 d) between seed treatment and inoculation. However, our results for seed treatment effects of mefenoxam are in accordance with Rodriguez-Brljevich^48^, who reported that the application of A14918E (fludioxonil + azoxystrobin + thiabendazole + mefenoxam) had positive effects on maize plant height, chlorophyll fluorescence (and thus photosynthetic performance), as well as a lower incidence of *Fusarium* spp. 52 days after emergence. Future studies should examine whether other crop plants can be protected from similar fungal diseases using seed treatments with *J. mimosifolia* extract (augmented with mefenoxam) and long periods between treatment and inoculation.

Disease reduction exhibited by the seed treatments was 20–26% higher in the pot experiment than in the field experiment, where other environmental factors modify the effects of biofungicide in addition to the plethora of microbes in the rhizosphere. The pot experiment was conducted under controlled conditions where the sole effect of the plant extract could be determined, whereas in the rhizosphere, soil microbes can degrade/utilize the components of the plant extract, resulting in a lower magnitude of the effect. Neem leaf extracts have been found to be effective against *F*. *verticillioides* and *Colletotrichum graminicola*^52^, giving > 90% reduction in seed-borne infection of *Curvularia lunata* and *Bipolaris sorghicola*. Abo-Elyousr et al.^53^, Mandal et al.^54^ and Rawal and Thakore^55^ also reported the substantial inhibition potential of *D. stramonium* leaf extracts against *F. solani*, which causes *Fusarium* rot of sponge gourd (*Luffa aegyptiaca*).

The expression of each of the genes investigated significantly correlated with the observed reduction in lesion length*.* Activation of defense responses, such as accumulation of PR-1, PR-5 and PR-10 transcripts in *F. verticillioides-*infected maize, has been reported in other studies^56–59^.

PR proteins and defense-related enzymes were analyzed to know more about the interaction of *F. verticillioides* with maize after the exogenous applications of *J. mimosifolia* extracts. In both experiments (pot and field) *J. mimosifolia* in combination with half-strength mefenoxam was the most effective treatment in increasing the activities of defense enzymes. These results reveal the positive effects of exogenous *J. mimosifolia* applications to enhance resistance in maize against *F. verticillioides* infections.

Exogenous application of specific plant extracts can induce resistance in the host plant via induction of higher levels of host defense enzymes and PR-proteins. This suggests that the active compounds present in the extracts induce systemic resistance in the host plants, resulting in reduction of disease development^60^. In our study, half-dose seed treatments with *J. mimosifolia* extracts with half-strength mefenoxam significantly increased the expression of genes encoding defense enzymes and PR-proteins; i.e. peroxidases, polyphenol oxidase, chitinase, PAL, glucanase, LTP, PR-1, PR-5 and PR-10, in response to *F. verticillioides* infection. These findings are in accordance with those of Campos-Bermudez et al.^61^, who reported an increase in the expression of glucanase and PR proteins in maize in response to *F. verticillioides.* The onset of induced systemic resistance in plants correlates with the increased activity and expression of PR proteins such as chitinases, β-1,3-glucanases, PAL and peroxidases; consequently, PR proteins are generally used as the key ingredients of systemic acquired resistance (SAR), an inducible immune response of plants that averts further infection or disease spreading to the noninfected parts of the host^62–64^.

Plant extracts have been reported to induce systemic resistance in host plants, disease reduction and increased plant growth in many agricultural crops^65,66^. When activated by various factors, plant defense genes that are quiescent in healthy uninoculated plants can induce systemic resistance against disease. Higher levels of POD, PPO, and PAL enzyme activities^65,67^, as well as expression of β-1,3-glucanase and chitinase genes^66,68,69^ have been reported to be effective against various fungal diseases. Lanubile et al.^70^ published a comprehensive review of genes in maize involved in the recognition of pathogens, the defense signaling network, and the activities of enzymes that contribute to host (maize) resistance against *F. verticillioides*.

Half-dose seed treatments with *J. mimosifolia* extract with half-strength mefenoxam provided defense in maize plants against stalk rot pathogen by inducing and upregulating the levels of PR-proteins, which may play an important role in strengthening the cell walls of the host plant to resist *F. verticillioides* infection. Pot and field experiments showed that the induced defense mechanism in response to plant extract applications was also detected in the protein profile revealed by SDS-PAGE. Polypeptide bands of 5, 37, 38 and 48 kDa were observed for the different treatments of *J. mimosifolia*, suggesting that these plant extract applications may provide resistance by inducing expression of specific proteins following pathogen inoculation. Hammond-Kosack et al.^71^ reported several defense-related proteins detected via SDS-PAGE. Proteins with molecular weights 14, 29, 33 and 45 kDa indicated the observed bands are likely to correspond to PR proteins^72^ but additional research (outside the scope of this study) is required to identify these proteins via mass spectrometry.

Plants are known to activate their defense pathways in response to pathogen inoculation via inducing new proteins to restrict the entry of the pathogen or its further spread^73^. The *J. mimosifolia* applications in combination with mefenoxam showed the maximum protein band densities in SDS-PAGE by enhancing the expression of pathogenesis-related proteins, as compared to the infected control, which showed the lowest band densities (Fig. 5). In both pot and field experiments, total soluble proteins and band densities of proteins showed a positive correlation which further confirmed the induction and/or upregulation of pathogenesis-related proteins (Supplementary Fig. 1). These findings are in accordance with Song et al.^74^ who also reported a positive correlation of total soluble protein with the band density values of SDS-PAGE.

In conclusion, the current research demonstrated that *J. mimosifolia* formulations under in vivo experiments efficiently controlled Fusarium stalk rot infection in maize. Leaf extracts of *J. mimosifolia* in combination with mefenoxam decreased the dose of fungicide required for *F. verticillioides* infection and induced resistance in the host plant via upregulation and accumulation of PR-proteins and defense enzyme activities. The mefenoxam cost in the present study applied to effectively control stalk rot disease is $13/ha (calculation based on the experiments conducted under field conditions), whereas the half-dose applications of *J. mimosifolia* with mefenoxam replaces 50% of the mefenoxam cost. The approximate cost for leaf extract formulation preparation might be around $1.2–2.5/ha because *J. mimosifolia* is a commonly grown tree. Hence application of leaf extracts of *J. mimosifolia* in combination with synthetic fungicide is financially promising. Moreover such biological fungicide formulations can not only minimize the harmful effets of synthetic fungicide but also protect human health and the environment from their residual effects. Future research should include further fractionation and detailed chemical analysis of the *J. mimosifolia* fractions (as begun in^75^) to determine the active compounds responsible for the protection of maize as demonstrated here.

## Supplementary information

Supplementary Information

## Data Availability

All the data is contained in the manuscript.
