# Supplementary material for: Induction of defense-related enzymes and enhanced disease resistance in maize against Fusarium verticillioides by seed treatment with Jacaranda mimosifolia formulations
Source: Sci Rep. 2021 Jan 8;11:59. doi: 10.1038/s41598-020-79306-x (PMC7794358; doi:10.1038/s41598-020-79306-x)

Induction of defense-related enzymes and enhanced disease resistance in maize against *Fusarium verticillioides* by seed treatment with *Jacaranda mimosifolia* formulations

Rabia Naz[1*], Asghari Bano[2], Asia Nosheen[1], Humaira Yasmin[1], Rumana Keyani[1], Syed Tahir Abbas Shah[1], Zahid Anwar[3] and Thomas H. Roberts[4*]

[1]Department of Biosciences, COMSATS University, Park Road, Chak Shahzad, Islamabad, Pakistan

[2]Department of Biosciences, University of Wah, Wah Cantt, Pakistan

[3]Department of Computer Science, COMSATS University Islamabad, Vehari campus, Pakistan

[4]Plant Breeding Institute, Sydney Institute of Agriculture, University of Sydney, NSW 2006 Australia.

Corresponding authors*

Rabia Naz

Department of Biosciences, COMSATS University, Park Road, Chak Shahzad, Islamabad, Pakistan

Phone: +92-51-90496086

Email: rabia.naz@comsats.edu.pk

Thomas H. Roberts
Plant Breeding Institute, Sydney Institute of Agriculture, University of Sydney, NSW 2006 Australia
Phone: +61-2-8627 1042
Fax: +61-2-8627 1099
Email: thomas.roberts@sydney.edu.au

Keywords: Disease control and pest management; Biochemistry and cell biology

Supplementary Table 1. Gene-specific primers used for RT-qPCR analysis

**Gene**	**Left primer**	**Right primer**	**Reference**
Chitinase	CCTCGCGAAAAGTAAACCAA	CATGCTGCAAACGGAAAGTA	Campos-Bermudez et al. (2013)
Glucanase	GCCTCTTCTACGGCAACAAG	AGTTAATTGCACCGCTCCTC	Campos-Bermudez et al. (2013)
LTP	GTACAAGAGGAACCCCAGCA	GCCCAAAGGTGCTCAAGTAG	Campos-Bermudez et al. (2013)
PR-1	CTCCAAACCCCACATTTGAT	GGAACGGTCCTGCTTGTTAC	Yuan et al. (2013)
PR-5	GTGCCCCAAGGGCGGGCCG	CTTCAAGGTTGGGAATTAAT	Yuan et al. (2013)
PR-10	CATTGGACTGGGATGAGCTT	CCACACAGAAAACCATGACG	Campos-Bermudez et al. (2013)
Actin 1	CTTCGAATGCCCAGCAAT	CGGAGAATAGCATGAGGAAG	Yuan et al. (2013)

Supplementary Table 2. General linear mixed model (GLMM) analysis for various parameters of maize in the pot experiment

Variables	Source	SS	DF	MS	*F*	*p*-value
Lesion Length (cm)	Treatments	5252.923	8	656.615	658.078	0.000
	Experiment	3.630	1	3.630	3.638	0.064
	Treatments * Experiment	77.480	8	9.685	9.707	0.000
Disease Reduction (%)	Treatments	21419.155	8	2677.394	1613.428	0.000
	Experiment	35.042	1	35.042	21.117	0.000
	Treatments * Experiment	28.087	8	3.511	2.116	0.060
Protein content	Treatments	1180169.708	11	107288.155	922.578	0.000
	Experiment	8085.681	1	8085.681	69.529	0.000
	Treatments * Experiment	587.486	11	53.408	.459	0.919
Chlorophyll content	Treatments	533.917	6	88.986	77.863	0.000
	Experiment	59.524	1	59.524	52.083	0.000
	Treatments * Experiment	9.560	6	1.593	1.394	0.252
Phenolic content	Treatments	14.780	6	2.463	645.616	0.000
	Experiment	0.132	1	0.132	34.666	0.000
	Treatments * Experiment	0.045	6	0.007	1.947	0.108
Acid invertase	Treatments	60.514	6	10.086	189.292	0.000
	Experiment	1.530	1	1.530	28.707	0.000
	Treatments * Experiment	0.078	6	0.013	.244	0.958
Protease	Treatments	282.090	6	47.015	242.173	0.000
	Experiment	4.378	1	4.378	22.551	0.000
	Treatments * Experiment	3.005	6	0.501	2.580	0.041
PPO activity	Treatments	12.134	6	2.022	29.421	0.000

	Experiment	1.355	1	1.355	19.713	0.000
	Treatments * Experiment	0.661	6	0.110	1.602	0.184
Peroxidase activity	Treatments	5.658	6	.943	55.623	0.000
	Experiment	1.106	1	1.106	65.223	0.000
	Treatments * Experiment	0.053	6	0.009	0.520	0.788
Chitinase activity	Treatments	73.131	6	12.188	264.209	0.000
	Experiment	2.069	1	2.069	44.860	0.000
	Treatments * Experiment	0.277	6	0.046	1.002	0.443
PAL activity	Treatments	4356.973	6	726.162	205.272	0.000
	Experiment	16.033	1	16.033	4.532	0.042
	Treatments * Experiment	3.633	6	0.606	0.171	0.982

Supplementary Table 3. General linear mixed model (GLMM) analysis for various parameters of maize in field experiment

Variables	Source	SS	DF	MS	*F*	*p*-value
Lesion Length (cm)	Treatments	5398.108	8	674.764	555.107	0.000
	Experiment	30.676	1	30.676	25.236	0.000
	Treatments * Experiment	9.006	8	1.126	.926	0.507
Disease Reduction (%)	Treatments	16754.333	8	2094.292	2233.249	0.000
	Experiment	33.765	1	33.765	36.005	0.000
	Treatments * Experiment	8.890	8	1.111	1.185	0.335
Protein content	Treatments	894397.153	11	81308.832	450.707	0.000
	Experiment	16050.347	1	16050.347	88.970	0.000
	Treatments * Experiment	1483.486	11	134.862	0.748	0.688
Chlorophyll content	Treatments	836.798	6	139.466	124.680	0.000
	Experiment	58.127	1	58.127	51.964	0.000
	Treatments * Experiment	7.186	6	1.198	1.071	0.403
Phenolic content	Treatments	12.498	6	2.083	107.584	0.000
	Experiment	0.526	1	0.526	27.176	0.000
	Treatments * Experiment	0.058	6	0.010	0.499	.804
Acid invertase	Treatments	43.461	6	7.243	263.649	0.000
	Experiment	1.241	1	1.241	45.176	0.000
	Treatments * Experiment	0.223	6	0.037	1.351	0.268
Protease	Treatments	818.930	6	136.488	106.199	0.000
	Experiment	10.450	1	10.450	8.131	0.000
	Treatments * Experiment	1.269	6	0.212	0.165	0.984
PPO activity	Treatments	7.354	6	1.226	30.646	0.000

	Experiment	1.475	1	1.475	36.872	0.000
	Treatments * Experiment	0.382	6	0.064	1.594	0.186
Peroxidase activity	Treatments	23.649	6	3.942	120.411	0.000
	Experiment	1.353	1	1.353	41.319	0.000
	Treatments * Experiment	0.280	6	.047	1.424	0.241
Chitinase activity	Treatments	145.243	6	24.207	0.618	0.714
	Experiment	81.195	1	81.195	2.072	0.161
	Treatments * Experiment	209.243	6	34.874	0.890	0.516
PAL activity	Treatments	5401.426	6	900.238	1213.414	0.000
	Experiment	67.894	1	67.894	91.513	0.000
	Treatments * Experiment	1.066	6	0.178	0.239	0.960

Supplementary Table 4. Numerical data supporting Fig. 2A (Heat map). The effect of *J. mimosifolia* formulations on relative expression profiles of selected defense-related genes 72 h after inoculation of maize with *F. verticillioides* in the pot experiment. Relative transcript abundance was determined using RT-qPCR. Data are expressed as mean ± standard error of three biological replicates. See Table 1 for abbreviations.

Treatments	Glucanase	Chitinase	LTP	PR-10	PR-1	PR-5
			**Year 1 (Experiment 1)**			
Fv	1.18±0.04	2.74±0.07	2.69±0.11	1.46±0.17	1.39±0.02	2.81±0.56
Mef+Fv	1.82±0.08	7.39±0.03	4.68±0.61	3.97±0.09	4.62±1.14	5.94±0.08
Jm+Fv	3.57±0.06	9.17±0.06	8.93±1.14	8.83±0.06	7.91±1.73	15.67±2.32
SA+Fv	3.30±0.12	11.63±0.14	6.14±1.73	6.54±0.56	9.73±1.13	9.89±0.04
½(Jm+Mef) + Fv	7.81±1.71	31.53±2.29	21.93±1.23	24.5±2.53	27.17±2.19	44.96±2.53
½(Jm+SA) +Fv	5.58±0.09	15.90±2.73	9.19±0.68	9.01±1.18	12.85±2.06	9.01±1.18
			**Year 2 (Experiment 2)**			
Fv	1.19±0.03	2.93±0.09	2.74±0.62	1.47±0.04	1.45±0.01	2.92±0.05
Mef+Fv	1.85±0.09	7.47±0.08	4.77±1.14	3.98±0.58	4.68±0.02	6.01±0.58
Jm+Fv	3.61±0.13	9.24±0.05	9.05±1.11	8.90±0.08	7.96±0.08	15.84±1.23
SA+Fv	3.36±0.03	11.84±1.19	6.17±0.55	6.64±0.19	9.80±1.81	10.06±0.07
½(Jm+Mef) + Fv	7.93±0.06	33.50±1.58	22.74±0.23	26.57±2.98	30.01±1.57	46.11±4.94
½(Jm+SA) +Fv	5.77±0.11	18.20±2.89	9.33±1.13	9.13±2.70	14.80±1.74	20.44±1.95

Supplementary Table 5. Numerical data supporting Fig. 2B (Heat map). The effect of *J. mimosifolia* formulations on relative expression profiles of selected defense-related genes 72 h after inoculation of maize with *F. verticillioides* in the field experiment. Relative transcript abundance was determined using RT-qPCR. Data are expressed as mean ± standard error of three biological replicates. See Table 1 for abbreviations.

Treatments	**Glucanase**	**Chitinase**	**LTP**	**PR-10**	**PR-1**	**PR-5**
			**Year 1**			
Fv	1.13±0.24	2.78±0.12	2.66±0.03	1.38±0.14	1.39±0.12	2.66±0.05
Mef+Fv	1.78±0.07	6.24±0.60	4.13±0.59	2.68±0.59	3.80±0.10	4.74±0.54
Jm+Fv	3.16±0.06	9.74±1.19	8.60±0.52	7.75±0.55	7.98±1.12	11.00±0.64
SA+Fv	3.06±0.66	11.93±1.41	5.50±1.69	4.86±1.13	8.75±0.62	9.60±1.13
½(Jm+Mef) + Fv	7.47±1.11	28.14±2.32	20.23±1.15	24.76±1.68	24.73±1.77	42.33±2.14
½(Jm+SA) +Fv	5.50±1.18	15.62±1.81	8.78±0.55	8.95±0.96	8.74±0.63	17.98±1.42
			**Year 2**			
Fv	1.18±0.05	2.85±0.15	2.73±0.53	1.55±0.10	1.43±0.26	2.70±0.50
Mef+Fv	1.73±0.55	6.35±0.55	4.21±0.17	2.77±0.11	3.94±1.13	4.81±0.48
Jm+Fv	3.21±0.57	9.93±0.59	8.87±1.14	7.95±1.72	8.74±0.31	11.17±0.51
SA+Fv	3.21±0.15	11.74±1.12	5.67±0.10	5.05±0.61	8.88±1.12	9.72±0.57
½(Jm+Mef) + Fv	7.6±0.66	28.21±1.68	20.35±1.69	25.14±1.49	44.09±1.81	25.20±1.28
½(Jm+SA) +Fv	5.59±0.22	15.69±1.26	9.00±1.17	9.25±1.17	8.89±0.67	18.04±1.20

Supplementary Table 6. The effect of different concentrations of methanolic extracts of *J. mimosifolia* on growth of maize seedlings in the petri plate experiment under axenic conditions. The data represents mean of three replicates.

**Treatments**	**Germination (%)**	**VI**	**Root L**	**Shoot L**	**Root FW**	**Root DW**	**Shoot FW**	**Shoot DW**
Control	85.3±3.9	21.6±1.2	19.7±1.12	8.7±0.44	0.14±0.005	0.019±0.001	0.29±0.014	0.019±0.002
J. mimosifolia (0.8%)	87.0±4.3	20.5±2.3	17.3±0.36	10.2±0.89	0.23±0.011	0.02±0.002	0.42±0.021	0.028±0.004
J. mimosifolia (1.0%)	86.0±1.9	23.54±1.31	18.5±1.38	17.5±1.14	0.24±0.012	0.023±0.002	0.59±0.027	0.035±0.006
J. mimosifolia (1.2%)	88.0±3.8	25.56±2.12	19.8±1.95	18.0±1.21	0.29±0.019	0.033±0.005	0.64±0.032	0.052±0.011

Supplementary Table 7. Effect of different concentrations of aqueous extracts of *J. mimosifolia* on growth of maize seedlings in the petri plate experiment under axenic conditions. The data represents mean of three replicates.

**Treatments**	**Germination (%)**	**VI**	**Root L**	**Shoot L**	**Root FW**	**Root DW**	**Shoot FW**	**Shoot DW**
Control	88.7±5.7	23.6±1.4	20.5±0.29	16.0±0.58	0.27±0.012	0.023±0.003	0.63±0.026	0.037±0.003
J. mimosifolia (0.8%)	90.0±5.1	22.3±1.77	19.5±0.25	9.83±0.44	0.16±0.009	0.03±0.003	0.313±0.013	0.023±0.003
J. mimosifolia (1.0%)	87.0±0.00	25.98±0.22	20.7±0.14	18.3±0.54	0.26±0.009	0.033±0.003	0.683±0.018	0.047±0.003
J. mimosifolia (1.2%)	90.0±5.1	27.02±1.42	20.7±0.18	18.6±0.35	0.34±0.021	0.04±0.006	0.703±0.024	0.060±0.006

VI= vigor index, RL= root length, SL= shoot length, FW= fresh weight, DW= dry weight

Supplementary Figure 1. Linear regression analysis between total soluble protein (X) and band density values from SDS-PAGE (Y) in **A:** pot experiment **B:** field experiment

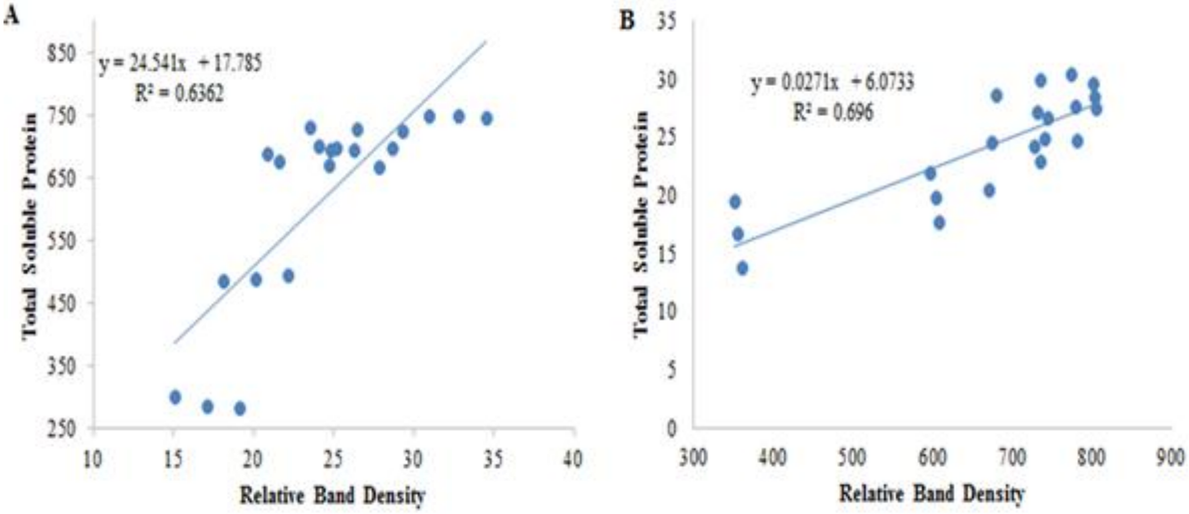

Supplementary Figure 2. The effect of *J. mimosifolia* aqueous extract and salicylic acid (SA) on the physiology of maize plants in the pot experiment

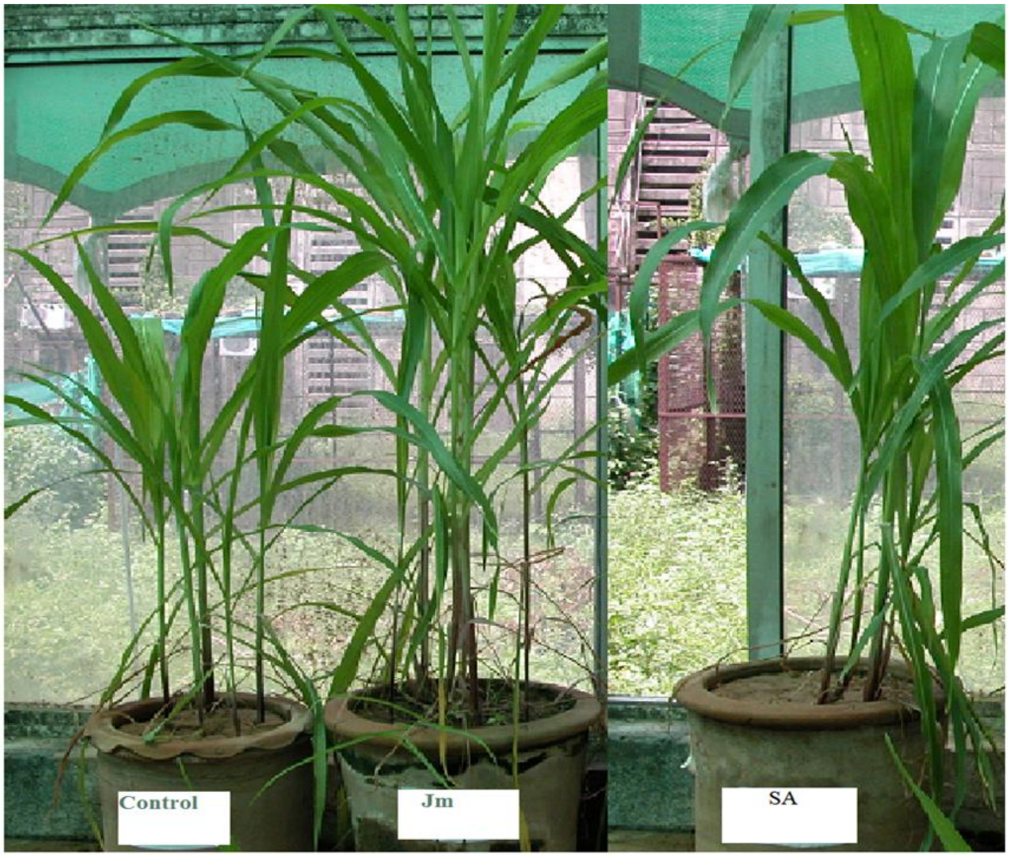

Supplementary Figure 3. The effect of *J. mimosifolia* methanolic extract and salicylic acid (SA) on the physiology of maize seedlings in the petri plate experiment under axenic conditions

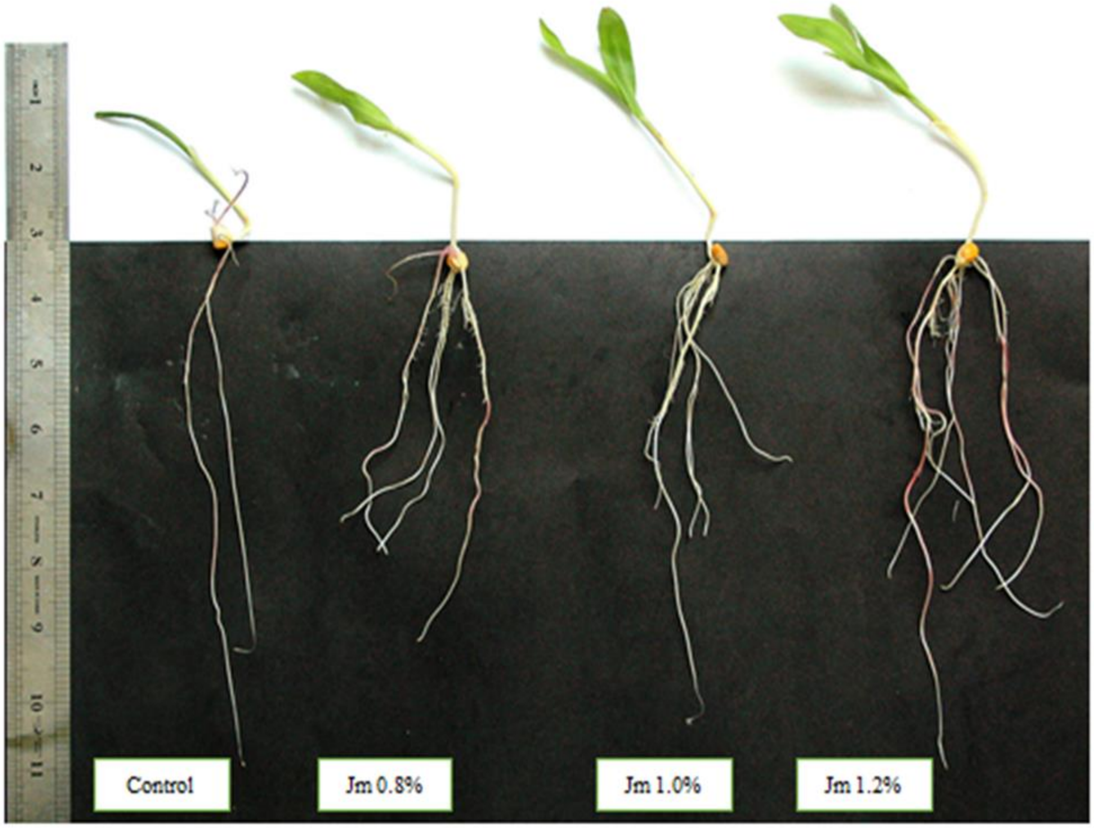

Supplementary Figure 4. The effect of *J. mimosifolia* aqueous extract and salicylic acid (SA) on the physiology of maize seedlings in the petri plate experiment under axenic conditions

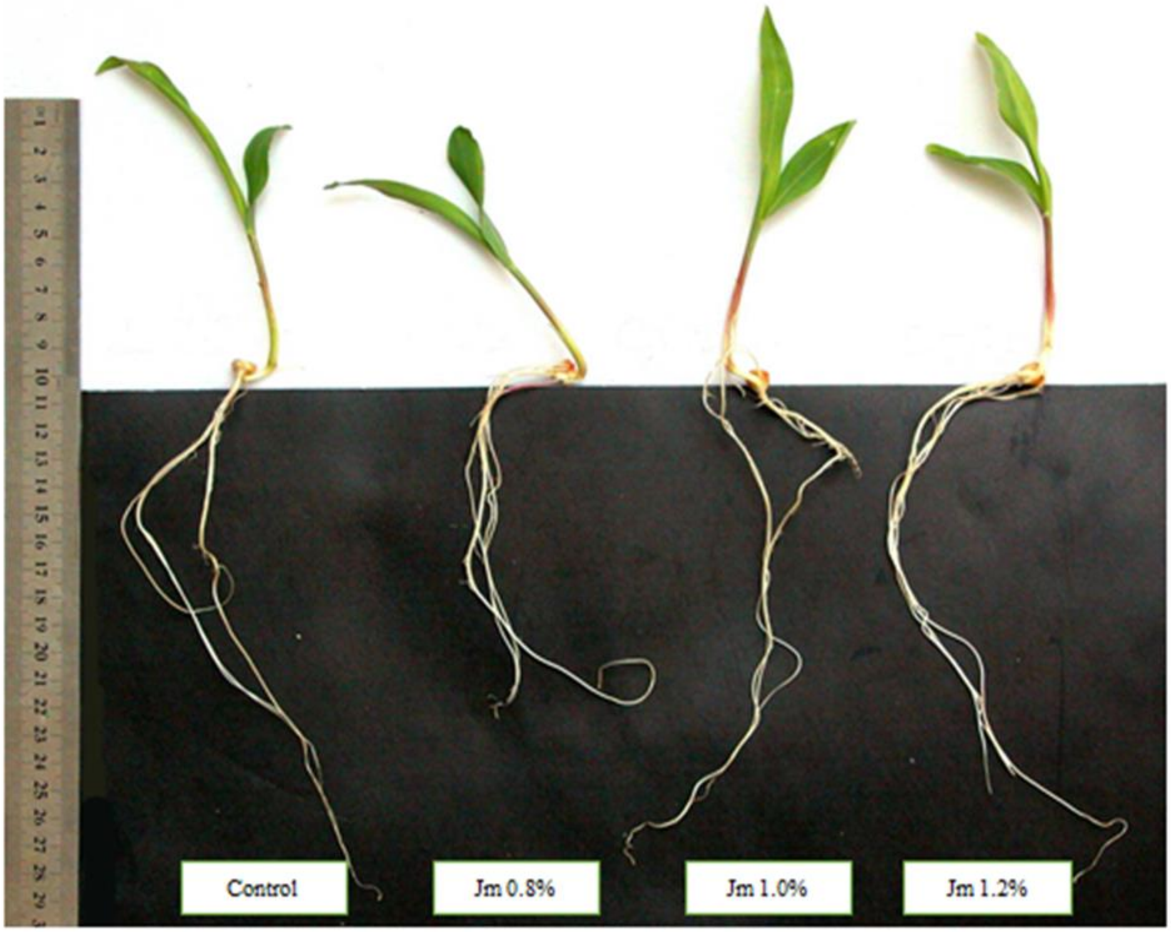

Supplement: Supplementary file 1 — Supplementary Information [file 41598_2020_79306_MOESM1_ESM.pdf]
